# Post-traumatic stress status among emergency department nurses during the winter H1N1 influenza season and correlations with burnout, coping, resilience, and support

**DOI:** 10.3389/fpsyg.2025.1514330

**Published:** 2026-06-16

**Authors:** Zheping Zheng, Dandan Shen, Yingqun Shi, Ling Chai, Yuhong Zhou

**Affiliations:** Shanghai Public Health Clinical Center Emergency Department, Shanghai, China

**Keywords:** emergency department nurses, post-traumatic stress disorder, occupational burnout, psychological resilience, coping styles, social support

## Abstract

**Objective:**

The H1N1 influenza, a highly contagious acute respiratory disease, causes a significant burden on public health, especially during the winter season when the number of cases surges. Emergency department nurses, who are on the frontline of patient care during this period, are exposed to high - stress situations, increasing their risk of developing post - traumatic stress disorder (PTSD). Understanding the status of PTSD among them and its influencing factors is crucial for safeguarding their mental health and ensuring quality healthcare. This study aims to investigate the status of post-traumatic stress disorder (PTSD) among emergency department nurses in multiple tertiary hospitals in Shanghai, China during the winter H1N1 influenza season and analyze its influencing factors.

**Methods:**

A cross - sectional study design was adopted from October 2023 to December 2023. A total of 116 emergency department nurses from multiple tertiary hospitals in Shanghai were selected as research subjects using cluster sampling during the peak period of winter H1N1 influenza. The Impact of Event Scale-Revised (IES-R), Maslach Burnout Inventory (MBI), Simplified Coping Style Questionnaire (SCSQ), Connor-Davidson Resilience Scale (CD-RISC), and Social Support Rating Scale (SSRS) were used for data collection. PTSD was defined as IES-R score ≥ 35, and participants were grouped as PTSD positive or negative. The relationship between PTSD and occupational burnout, coping styles, psychological resilience, and social support was analyzed.

**Results:**

Among the 116 participants, the rate of PTSD positivity was 44.86% (48/107). The MBI scores in all dimensions were significantly higher in the PTSD positive group compared to the PTSD negative group. The positive coping scores in the SCSQ were lower in the positive group, while the negative coping scores were higher. The CD-RISC scores in all dimensions were significantly lower in the PTSD positive group, as were the SSRS scores in all dimensions. Higher emotional exhaustion was significantly associated with increased odds of PTSD (OR = 1.16), indicating that for each one-point increase in emotional exhaustion, the odds of PTSD increased by 16%. Greater reliance on negative coping strategies was also independently associated with PTSD (OR = 1.23). In contrast, higher levels of optimism (a resilience factor) and greater subjective social support were protective, significantly lowering the odds of PTSD (OR = 0.76 and OR = 0.70, respectively).

**Conclusion:**

The occurrence of PTSD among emergency department nurses during the winter H1N1 influenza season is considerable and closely related to occupational burnout, psychological resilience, coping styles, and social support. These findings highlight the urgent need for attention to this issue. However, this study has several limitations. The sample was only from top - tier hospitals in Shanghai during the winter influenza season, which may limit the generalizability of the findings. Also, relying on self-reported questionnaires may be subject to recall bias and self - reporting subjectivity.

## 1 Introduction

The H1N1 influenza, caused by the H1N1 influenza virus, is a highly contagious acute respiratory disease with a seasonal prevalence. It poses a substantial burden on public health, leading to high hospitalization rates and significant morbidity and mortality (Moghadami, 2017). The winter months are characterized by a peak in H1N1 influenza cases, particularly affecting vulnerable populations such as children and the elderly, resulting in a considerable disease burden globally. From the 2010–2011 to 2013–2014 influenza seasons, influenza-related respiratory and circulatory deaths ranged from 12,000 to 56,000, and from 4,000 to 12,000 in the six seasons from 2010–2011 to 2015–2016 (Rolfes et al., 2018). The H1N1 influenza epidemic has caused an unexpected surge in morbidity and mortality rates, thereby increasing the demand for healthcare services (Losier et al., 2023). Consequently, there has been an intensified strain on healthcare systems due to the rapid influx of patients, resulting in a decreased nurse-to-patient ratio and an elevated workload (Jia et al., 2023). The COVID-19 pandemic has shed light on the mental health challenges faced by healthcare workers, revealing a higher prevalence of post-traumatic stress disorder (PTSD) among individuals working long hours, in isolation wards, and particularly among female nurses (Qi et al., 2022).

The emergency department serves as a critical gateway for admitting and providing care to critically ill patients. Due to the unique nature of their work and the environment they operate in, emergency department nurses are frequently exposed to traumatic or stressful events, placing them under greater psychological pressure compared to other groups and making them more susceptible to physical and mental health issues (Caruso et al., 2021). During the winter H1N1 influenza season, the overwhelming influx of patients places significant strain on the healthcare system, particularly impacting emergency department nurses (Schuster and Dwyer, 2020). PTSD is a prevalent psychological distress experienced by emergency department nurses following exposure to highly stressful events. The nature of emergency nursing involves caring for patients with diverse medical conditions, many of whom are critically ill, have complex diseases, and require urgent treatment. Emergency nursing staff often work under heavy workloads for prolonged periods without adequate time to recover physically and mentally. This continuous exposure to traumatic events in the workplace can trigger the development of mental symptoms, leading to the onset of PTSD (Winnand et al., 2023). The consequences of PTSD among emergency department nurses are far-reaching, as it can diminish their caregiving abilities, increase the occurrence of nursing errors, and amplify the desire to leave the profession, thereby impacting the overall quality and safety of clinical care. Consequently, conducting risk assessments and implementing early interventions for PTSD among emergency department nurses are of significant importance (Ramachandran et al., 2023).

Occupational burnout, coping style, psychological resilience and social support are important factors affecting mental health. The characteristics of high intensity, high pressure and high risk of clinical nursing work make nurses a high-risk group of occupational burnout (Kaplan, 2021). Burnout, coping styles, resilience and social support are important factors affecting mental health. The high intensity, high pressure and high-risk characteristics of clinical nursing work make nurses a high-risk group of job burnout (Sipos et al., 2024). Although numerous studies have focused on the mental health of healthcare workers, research on post-traumatic stress disorder (PTSD) and its overall relationship to burnout, coping styles, resilience, and social support among nurses in emergency departments of tier 1 hospitals in Shanghai remains inadequate for this particular period of the winter H1N1 flu season. Most of the existing studies focus on the impact of ordinary periods or single factors on nurses’ mental health, and lack in-depth analysis of the combined effect of multiple factors, especially during specific periods of high incidence of influenza and sudden increase in work pressure. Based on the above background, this study aims to explore the following key questions: (1) What is the current status of PTSD among emergency department nurses in multiple tier 1 hospitals in Shanghai during the winter H1N1 influenza season? (2) What are the relationships between factors such as burnout, coping styles, mental resilience, and social support and PTSD among emergency department nurses? (3) How can targeted interventions be developed based on these relationships to improve the mental health of emergency department nurses and improve the quality of nursing services?

## 2 Methods

### 2.1 Study design

The study design is described as a cross-sectional study using cluster sampling during the peak of the winter H1N1 influenza season from October 1, 2023 to December 1, 2023. A cluster sampling method was used to select 116 emergency department nurses from 6 tertiary hospitals in Shanghai (Located in different regions of Shanghai) during the peak season of winter H1N1 influenza. The participants were assessed using the Impact of Event Scale-Revised (IES-R), Maslach Burnout Inventory (MBI), Simplified Coping Style Questionnaire (SCSQ), Connor-Davidson Resilience Scale (CD-RISC), and Social Support Rating Scale (SSRS). Before enrolling, all participants were informed about the study and they provided written consent after being briefed on the details. This study was approved by the ethics committee of our hospital (No. 2020839).

### 2.2 Inclusion and exclusion criteria

Inclusion criteria: (1) Female nurses aged 20 to 60 years; (2) Practicing nurses in the emergency department who had been working in the department for at least 6 months before and after the survey; (3) Informed about the study design and willing to complete the relevant questionnaires; (4) On-the-job emergency nurse, working experience ≥ 1 year.

Exclusion criteria: (1) Significant family events or other circumstances affecting mental health within the past 3 months; (2) History of anxiety, depression, or other psychological disorders; (3) Stressful life events such as professional qualification examinations or children’s education within the 3 months before enrollment; (4) Special circumstances such as pregnancy or lactation.

### 2.3 Sampling methods and post-event efficacy testing

Two-stage cluster sampling was used to randomly select 6 tier 1 hospitals in Shanghai in the first stage, and nurses were randomly selected from the emergency department of each hospital in the second stage. The total number of nurses in the emergency department of each hospital is about 30–50, and the proportion is randomly sampled by 50%. Finally, 15–25 people were included in each hospital, and a total of 90 to 150 subjects were included.

Using the t-test method of two independent samples, the lowest Cohen’s d-effect size was known to be 0.47, and a total of 145 people were included. The first group had a sample size of n1 = 68, the second group had a sample size of n2 = 77, and the significance level was set to double-sided test *α* = 0.05. After the efficacy analysis was carried out by G * Power software, the final result was 0.80.

### 2.4 Questionnaire content

This study distributes questionnaires through a combination of online and offline methods. Online, through a specialized questionnaire platform, the questionnaire link is sent to the head nurses of the emergency departments of each participating hospital, and then the head nurses forward it to the nurses in the departments. Offline, trained researchers go to the emergency departments of each hospital and distribute paper questionnaires to nurses who meet the inclusion criteria during the nurses’ work breaks, and provide on-site guidance for filling them out.

#### 2.4.1 Impact of event scale-revised

The IES-R (Christianson and Marren, 2012) was used to assess the presence and severity of post-traumatic stress symptoms among the participants. The scale consists of 22 items that measure intrusion, avoidance, and hyperarousal symptoms. Each item is rated on a 5-point Likert scale, ranging from 0 (not at all) to 4 (extremely). A total score of 35 or higher was defined as a positive indication of PTSD, while a score below 35 indicated a negative indication of PTSD. The Cronbach’s *α* coefficients in this study for IES-R was 0.89 (α = 0.89).

#### 2.4.2 Maslach burnout inventory

The MBI (Dall'Ora et al., 2020) was employed to evaluate occupational burnout among the participants. The MBI comprises three components: emotional exhaustion, depersonalization, and personal accomplishment. Each component includes multiple items that individual’s rate on a 7-point Likert scale, ranging from 0 (never) to 6 (every day). Elevated scores on the emotional exhaustion and depersonalization sections signify increased burnout levels, whereas higher scores on the personal accomplishment segment suggest diminished burnout levels. The Cronbach’s *α* coefficients in this study for MBI was 0.82 (α = 0.82).

#### 2.4.3 Simplified coping style questionnaire

The SCSQ (Ma et al., 2022) was used to assess the participants’ coping styles. The survey comprises two dimensions: positive coping and negative coping. Each dimension comprises 20 items rated on a 4-point Likert scale, ranging from 1 (never) to 4 (always). Elevated scores on the positive coping dimension suggest a greater inclination toward positive coping strategies, whereas higher scores on the negative coping dimension indicate a stronger tendency toward negative coping strategies. The Cronbach’s *α* coefficients in this study for SCSQ was 0.76 (α = 0.76).

#### 2.4.4 Connor-Davidson resilience scale

The CD-RISC (Connor and Davidson, 2003) was utilized to measure the participants’ psychological resilience. The instrument consists of 25 items evaluating personal competence, high standards, and persistence. Each item is appraised on a 5-point Likert scale, spanning from 0 (not true at all) to 4 (true nearly all the time). Elevated scores signify increased levels of psychological resilience. The Cronbach’s *α* coefficients in this study for CD-RISC was 0.79 (α = 0.79).

#### 2.4.5 Social support rating scale

The SSRS (Zou et al., 2023) was used to evaluate the participants’ perceived social support. The measurement tool comprises three components: objective support, subjective support, and utilization of support. Each component includes multiple items rated on a 4-point Likert scale, varying from 1 (strongly disagree) to 4 (strongly agree). Elevated scores on every component indicate increased levels of perceived social support. The Cronbach’s *α* coefficients in this study for SSRS was 0.81 (α = 0.81).

Demographic information of the participants was also collected, including age, gender, years of work experience, educational level, professional title, etc. At the same time, other relevant information that may affect the research results was recorded, such as whether they had participated in major training recently and their home location.

### 2.5 Statistical analysis

Descriptive statistics were employed to outline the demographic attributes of the participants. For continuous variables (such as age, years of work, etc.), they are described in the form of mean ± standard deviation (mean ± sd). For categorical variables (such as educational level and professional title), they are presented in the form of counts and percentages. To assess variances between the PTSD positive and negative groups, the chi-square test and t-test were utilized. To identify the independent predictors of PTSD symptoms among emergency department nurses, we conducted a supplementary multivariate regression analysis. Statistical computations were carried out using SPSS version 26.0 software (IBM Corp., Armonk, NY, United States) and GraphPad Prism 9.0 software (Dotmatics, Boston, Massachusetts, United States). Statistical significance was established for *p*-values below 0.05.

## 3 Results

### 3.1 Baseline profiles

Among the 161 subjects included in the study, a total of 145questionnaire results met the criteria. There were 68 cases of PTSD-positive and 77cases of PTSD-negative, resulting in a PTSD-positive rate of 46.90% (68/145). The general characteristics of the two groups are presented in Table 1. The baseline profiles were balanced in age, years of work experience, education level, or professional title between the two groups (all *p* > 0.05).

**Table 1:** Baseline profiles between 68 PTSD-positive and 77 PTSD-negative ED nurses.

Index	PTSD-positive group	PTSD-negative group	t/χ^2^	*p*
n	68	77		
Age ( $\bar{x}$ ±s, years)	32.02 ± 4.69	31.26 ± 5.09	0.428	0.796
Work Experience ( $\bar{x}$ ±s, years)	8.69 ± 2.06	9.11 ± 2.47	0.941	0.349
Education Level			3.337	0.068
College or Below	41	26		
Bachelor’s Degree or Above	27	33		
Professional Title			1.849	0.397
Nurse	34	46		
Senior Nurse	25	25		
Head Nurse	9	6		

### 3.2 Comparison of occupational burnout

MBI was utilized to evaluate occupational burnout among the participants (Table 2). Significantly higher MBI scores across all dimensions were observed in the PTSD-positive group compared to the PTSD-negative group (all *p* < 0.05). Emotional exhaustion index effect size d = 1.12, indicated that there is a large difference in this measure between the two groups. Depersonalization index effect size d = 0.73, indicated that there is a moderate difference in this measure between the two groups. Reduced personal accomplishment index effect size d = 0.74, indicated that there is a moderate difference in this measure between the two groups. The positive sign indicates that the mean value of the PTSD positive group is higher than those of the PTSD negative group.

**Table 2:** Occupational burnout between 68 PTSD-positive and 77 PTSD-negative ED nurses.

Group	N	Emotional exhaustion	Depersonalization	Reduced personal accomplishment
PTSD-positive group	68	34.58 ± 7.26	13.25 ± 3.28	38.29 ± 8.21
PTSD-negative group	77	27.11 ± 6.18	11.01 ± 2.85	32.60 ± 7.18
Cohen’s d		1.11	0.73	0.74
T		6.69	4.40	4.46
*p*		<0.001	<0.001	<0.001

### 3.3 Comparison of coping styles between PTSD-positive and PTSD-negative

**Figure 1:**
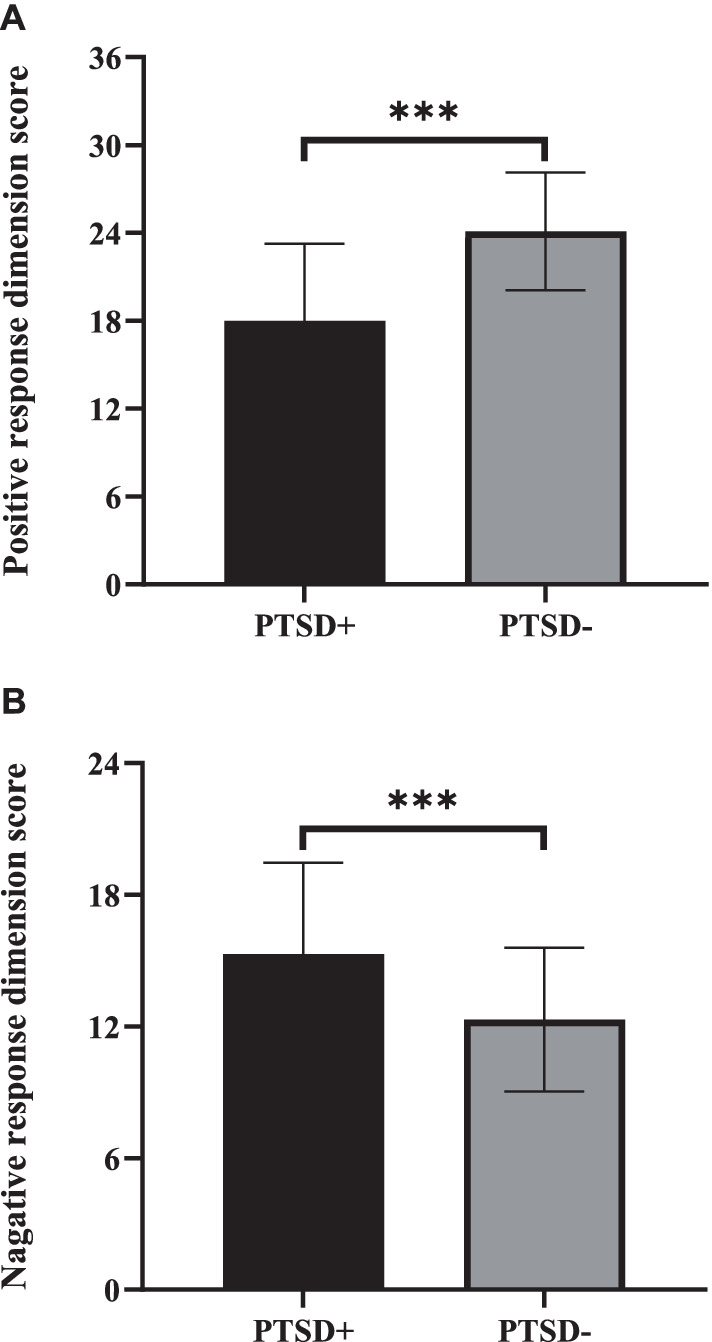
Coping styles between 68 PTSD-positive and 77 PTSD-negative ED nurses. ***indicated *p* < 0.001. **(A)** Positive coping styles in PTSD-positive and PTSD-negative nurses. This graph compares the scores for positive coping styles (e.g., problem-solving, seeking social support) between PTSD-positive and PTSD-negative emergency department nurses. The PTSD-positive group exhibited significantly lower scores (*p* < 0.001), indicating a reduced tendency to use adaptive coping strategies during the winter H1N1 influenza season. **(B)** Negative coping styles in PTSD-positive and PTSD-negative nurses. This graph illustrates the scores for negative coping styles (e.g., avoidance, self-blame) between PTSD-positive and PTSD-negative emergency department nurses. The PTSD-positive group showed significantly higher scores (*p* < 0.001), reflecting a greater reliance on maladaptive coping mechanisms under high-stress conditions.

SCSQ was used to assess the coping styles of the subjects, and the results are presented in Figure 1. The PTSD-positive group had lower scores for positive coping and higher scores for negative coping compared to the PTSD-negative group (all *p* < 0.05).

### 3.4 Comparison of psychological resilience

**Figure 2:**
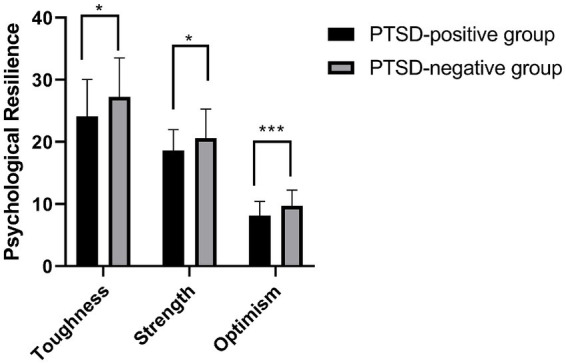
Psychological resilience between 68 PTSD-positive and 77 PTSD-negative ED nurses. *indicated *p* < 0.05, ***indicated *p* < 0.001.

CD-RISC was used to assess the psychological resilience of the subjects, and the results are presented in Table 3 and Figure 2. The CD-RISC scores in all dimensions were significantly lower in the PTSD-positive group compared to the PTSD-negative group (all *p* < 0.05). Toughness index effect size d = −0.52, Strength index effect size d = −0.47, Optimism index effect size d = −0.64, all indicating that there are moderate differences in these three indicators between the two groups. The negative sign indicates that the mean value of the PTSD positive group is lower than those of the PTSD negative group.

**Table 3:** Psychological resilience between 68 PTSD-positive and 77 PTSD-negative ED nurses.

Group	N	Toughness	Strength	Optimism
PTSD-positive group	68	24.11 ± 5.93	18.63 ± 3.36	8.11 ± 2.31
PTSD-negative group	77	27.26 ± 6.25	20.58 ± 4.71	9.68 ± 2.57
Cohen’s d		−0.52	−0.47	−0.64
t		−3.10	−2.84	−3.82
*p*		0.002	0.005	<0.001

### 3.5 Comparison of social support

**Figure 3:**
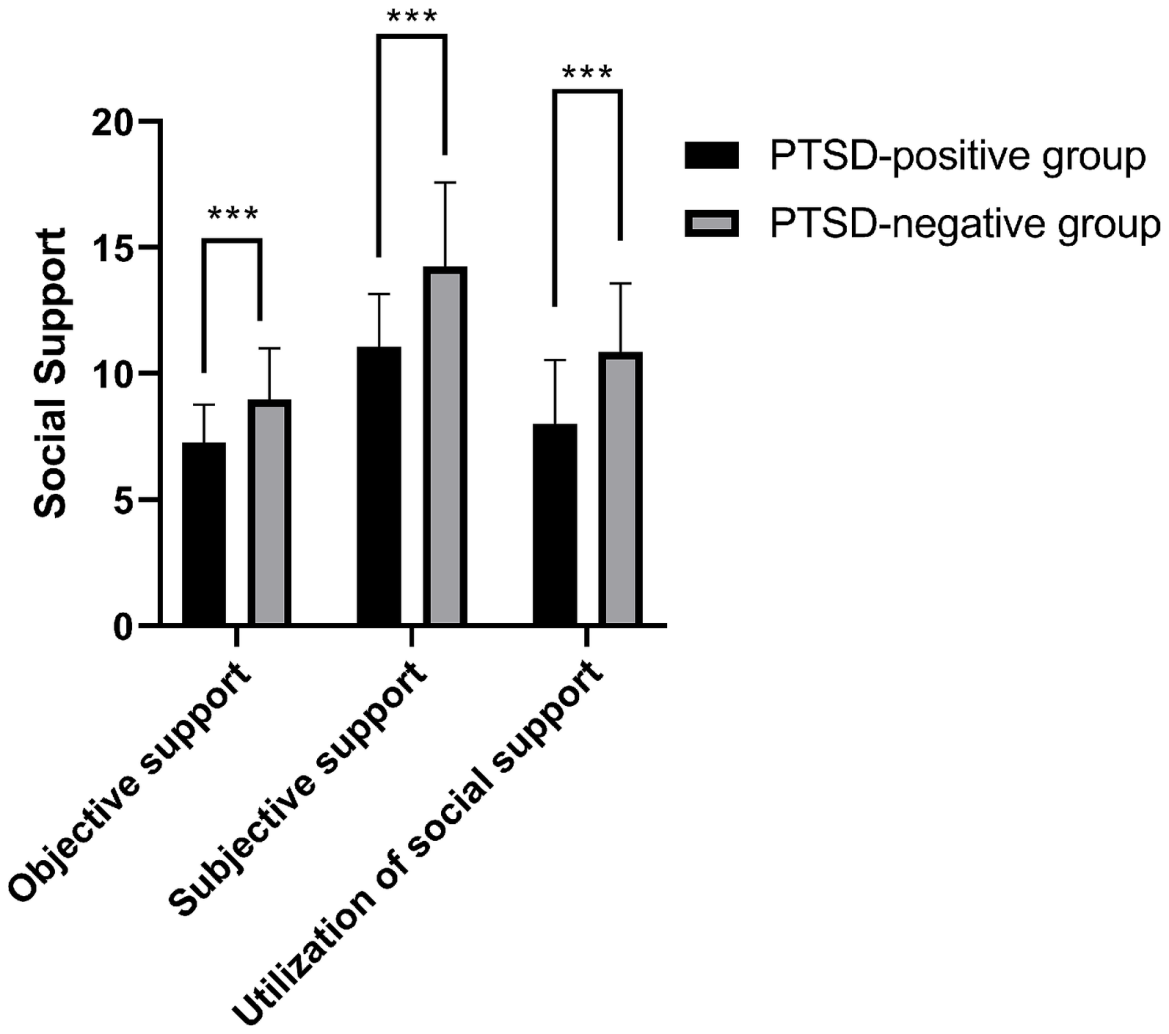
Social support between 68 PTSD-positive and 77 PTSD-negative ED nurses. ***indicated *p* < 0.001.

SSRS was employed to assess social support (Table 4; Figure 3). Notably, the SSRS ratings in all dimensions exhibited a significant decrease in the PTSD-positive group compared to the PTSD-negative group (all *p* < 0.05). Objective support index effect size d = −0.94, Subjective support index effect size d = −1.11, Utilization of social support index effect size d = −1.08, all indicating that there are large differences in these three indicators between the two groups. The negative sign indicates that the mean value of the PTSD positive group is lower than those of the PTSD negative group.

**Table 4:** Social support between 68 PTSD-positive and 77 PTSD-negative ED nurses.

Group	n	Objective support	Subjective support	Utilization of social support
PTSD-positive group	68	7.25 ± 1.52	11.08 ± 2.08	8.01 ± 2.53
PTSD-negative group	77	8.97 ± 2.03	14.24 ± 3.34	10.85 ± 2.72
Cohen’s d		−0.95	−1.12	−1.08
T		−5.71	−6.73	−6.46
*p*		<0.001	<0.001	<0.001

### 3.6 Logistic regression analysis of factors associated with PTSD

To further explore the independent predictors of PTSD among emergency department nurses, a multiple logistic regression model was performed. PTSD status (positive = 1, negative = 0) was used as the dependent variable. Variables included as independent predictors were those that showed significant differences in univariate analyses: emotional exhaustion, depersonalization, reduced personal accomplishment (MBI subscales), negative coping, positive coping (SCSQ), psychological resilience subscales (CD-RISC), and social support subscales (SSRS).

Demographic variables such as age, years of work experience, and education level were also entered as control covariates to adjust for potential confounding (Table 5).

**Table 5:** Multiple logistic regression predicting PTSD (*n* = 145).

Variable	β (SE)	OR (95% CI)	*p*-value
Emotional exhaustion (MBI)	0.15 (0.05)	1.16 (1.05–1.29)	0.004**
Negative coping (SCSQ)	0.21 (0.09)	1.23 (1.04–1.46)	0.017*
Optimism (CD-RISC)	−0.28 (0.11)	0.76 (0.62–0.93)	0.008**
Subjective social support (SSRS)	−0.35 (0.10)	0.70 (0.58–0.84)	<0.001**
Age	0.02 (0.05)	1.02 (0.93–1.13)	0.642
Years of work	−0.04 (0.07)	0.96 (0.83–1.10)	0.531

*Significant at *p* < 0.05; **Significant at *p* < 0.01.

## 4 Discussion

This research demonstrates that during the winter H1N1 flu season, the PTSD-positive screening rate among emergency department nurses in top tertiary hospitals reached an alarming 46.90% (68/145). Nurses frequently find themselves under duress and in extreme conditions as they tirelessly combat for their patients’ life and well-being, rendering them susceptible to stress and trauma (Kosydar-Bochenek et al., 2017). Emergency nursing staff often encounter distressing and traumatic events, such as accidents, violence, and death. Repeated exposure to such incidents can result in the manifestation of PTSD symptoms. A meta-analysis encompassing 30,878 research subjects revealed that the prevalence rates of PTSD, depression, anxiety, and overall psychological distress among emergency personnel were 11, 15, 15, and 27%, respectively (Petrie et al., 2018). A nationwide cross-sectional survey conducted in Germany discovered a significant decrease in happiness levels among nursing personnel, with PTSD and depression rates found to be higher compared to the general population (Eiche et al., 2019). The COVID-19 pandemic has brought attention to the psychological well-being of healthcare professionals, with frontline healthcare workers, including nursing staff, potentially experiencing severe and long-lasting psychological consequences. Research by Asadi Aghajari et al. suggests that one-third of emergency healthcare professionals are affected by PTSD, exhibiting significant correlations with depression and occupational stress (Asadi Aghajari et al., 2023). While the study aimed to assess PTSD symptoms among emergency department nurses during the peak of the winter H1N1 influenza season, it is important to acknowledge that several potentially influential confounding variables were not measured or controlled for in the study design. Although participants with “significant family events” were excluded, other important factors such as prior mental health history, financial stress, sleep disturbances, and shift type (e.g., night vs. day shifts) were not accounted for. These variables are known to significantly influence both psychological resilience and vulnerability to occupational stress. For example, nurses working extended or irregular night shifts may experience more severe disruptions to circadian rhythms, reduced recovery time, and increased psychological burden, all of which can elevate PTSD risk. The relatively high rate of reported PTSD symptoms in the study may therefore reflect not only the acute stress of working during a pandemic peak but also the cumulative effects of pre-existing stressors and individual risk factors that were not assessed. Future studies should aim to include a broader range of personal, occupational, and psychosocial variables to better isolate the impact of occupational exposure from other contributing factors. A more nuanced understanding of these confounders would improve the accuracy of PTSD prevalence estimates and inform more targeted interventions for at-risk healthcare workers.

The findings of this study reveal that individuals with a positive screening for PTSD exhibit significantly higher scores in all dimensions of the MBI compared to the PTSD-negative group. Furthermore, the positive coping scores in the SCSQ were lower in the PTSD-positive group, while the negative coping scores were higher. In addition, the CD-RISC scores in each dimension were significantly lower in the PTSD-positive group, as were the SSRS scores in each dimension. Burnout is characterized by emotional exhaustion, decreased sense of accomplishment, depersonalization, and indifference toward work, resulting from an enduring imbalance between work and rest. Clinical nursing, characterized by high intensity, technical complexity, and occupational hazards, ranks among the professions with the highest prevalence of burnout (Edú-Valsania et al., 2022). The demanding nature of the nursing profession, coupled with the challenging work environment, has led to persistently high rates of burnout among healthcare practitioners (Shanafelt et al., 2015; Shanafelt et al., 2022). Notably, Chirico et al. highlighted that burnout represents a more serious occupational health concern, with professionals in high workload occupations, such as teachers and healthcare providers, being particularly susceptible to burnout (Sullivan et al., 2022). The detrimental effects of burnout encompass chronic work-related stress, increased intent to leave the profession, and compromised quality of healthcare delivery and nursing care (Gualano et al., 2021). Previous research has demonstrated that burnout serves as a significant predictor for post-traumatic stress disorder symptoms among firefighters and police officers, with higher levels of burnout associated with more severe post-traumatic stress symptoms (Ogińska-Bulik and Juczyński, 2021; Jo et al., 2018).

Coping strategies play a crucial role in the prevention and management of PTSD. Positive coping strategies involve accepting reality, confronting stressors head-on, actively seeking solutions, and seeking support when needed. Conversely, negative coping strategies encompass distorting reality, avoiding self-reflection, and exhibiting self-destructive behaviors. Higher scores on negative coping strategies are associated with an increased vulnerability to post-traumatic stress reactions (Kucmin et al., 2018). The negative coping style of nurses was a more prominent predictor of PTSD than that of teachers, possibly related to the high-pressure environment in the emergency department (Zhang et al., 2019). In the aftermath of a traumatic event, it is advised that emergency department nurses who exhibit higher levels of negative coping strategies engage in open communication with colleagues, participate in community service activities, and employ humor or lightheartedness as a means to alleviate stress (Olff et al., 2025).

Psychological resilience refers to an individual’s capacity to effectively cope with and recover from adversity, as well as adapt to challenging circumstances (Sisto et al., 2019). Resilient individuals demonstrate flexibility in navigating various difficulties and challenges, and they possess the ability to adapt to uncertainty and change without succumbing to significant negative repercussions (Rakesh et al., 2019). According to the resource preservation theory, emergency nurses face resource depletion (such as work overload) during the flu season, while resilience (resource gain) reduces PTSD risk by buffering stress (Zhou et al., 2024). Psychological resilience serves as a pivotal factor in facilitating individuals’ recovery from stress and trauma, and it contributes to the prevention and mitigation of professional burnout among clinical nurses. Research has indicated that individuals with high levels of psychological resilience can ameliorate the adverse impact of prior traumatic experiences on the development and severity of PTSD symptoms (Cheng et al., 2023). Social support serves as a critical interpersonal resource that significantly influences the improvement of mental health outcomes following disasters (Calhoun et al., 2022). Insufficient social support exacerbates PTSD symptoms among nursing professionals who experience high levels of occupational stress (Hao et al., 2023). Unlike the burnout model from an evolutionary perspective (e.g., resource imbalance due to social class conflict), the direct protective effect of social support in this study is more significant, possibly due to the enhanced resource reserve of the medical team’s collaborative culture (Garcia, 2024). Chinese nurses rely more on colleagues to support stress relief, which is different from the predictive effect of family type (such as nuclear family) on PTSD in the Nepalese study, possibly reflecting the importance of teamwork in a collectivist culture (Parajuli et al., 2023).

In the context of frequent global public health incidents, the mental health of healthcare workers has attracted much attention. The findings of this study on a specific group of emergency department nurses during the winter H1N1 flu season not only enrich the content of mental health research of healthcare workers in specific departments during special periods in this field, but also provide reference for other similar studies, highlighting the importance and urgency of paying attention to the mental health of healthcare workers. In view of the higher incidence of PTSD among emergency department nurses in the winter H1N1 influenza season and related influencing factors in this study, it is necessary to strengthen the intervention measures for healthcare workers, which can improve the self-esteem of healthcare workers, promote psychological well-being and enhance stress coping skills. PTSD intervention for nurses can learn from postpartum trauma prevention strategies (such as expressive writing intervention), but group psychological training needs to be designed according to occupational characteristics (Dekel et al., 2024). In addition, programs that enhance psychological capital among teachers, such as resilience training, are equally applicable to nurses, but need to strengthen their coping skills in crisis situations (Du et al., 2022). The high comorbidity of PTSD (e.g., depression, anxiety) was also reflected in this study, suggesting the need for comprehensive intervention rather than single symptom management. As a complementary perspective, it is crucial to consider the psychological and occupational impacts faced by anesthesia professionals, particularly the prevalence of burnout syndrome. Recent literature has highlighted the significant emotional toll and chronic stress experienced by healthcare workers in high-stakes environments such as operating rooms. For instance, the study by Raudenská et al. (2020) explores the neurobiological underpinnings of burnout, emphasizing the role of chronic occupational stress in impairing clinical decision-making. Similarly, research published in the Revista Colombiana de Salud Ocupacional e Higiene Industrial (Nogueira Alves et al., 2023) analyzes burnout prevalence among healthcare providers in Latin America, suggesting systemic factors such as workload, support systems, and job control as key contributors. Most recently, a 2024 study in Frontiers in Psychology (Lin et al., 2024) further underscores the psychological vulnerability of healthcare professionals post-pandemic, linking emotional exhaustion with decreased patient care quality. Including these perspectives provides a more holistic understanding of the challenges faced in anesthetic practice and reinforces the need for institutional strategies to mitigate burnout and promote clinician well-being.

However, the limitations should be acknowledged. Sample selection limitation: The sample in this study only included emergency department nurses from top-tier hospitals during the winter influenza season, which may not represent nurses from other regions or different types of healthcare institutions. Therefore, the generalizability of the research findings may be limited. Recall bias: This study relied on self-reported questionnaires for data collection, which may be susceptible to recall bias. Nurses may have difficulty accurately recalling past events and experiences, which could affect their reporting of PTSD symptoms, burnout, psychological resilience, and other variables.

### 4.1 Limitation and recommendation for future research

One important limitation of this study is the absence of follow-up assessments. Given that PTSD symptoms can develop weeks or even months after exposure to traumatic events, the short data collection window may not fully capture the true incidence of PTSD among participants. As a result, cases of delayed-onset PTSD may have been missed. To address this limitation, future research should consider adopting a longitudinal study design, incorporating follow-up assessments at multiple time points (e.g., 3 months, 6 months post-exposure). This would allow for a more comprehensive understanding of the onset, progression, and persistence of PTSD symptoms over time, and would enhance the accuracy and clinical relevance of findings related to mental health outcomes among healthcare workers during epidemic or pandemic events.

### 4.2 Recommendation

Self-report measurement: The scales used in this study were based on self-reporting by the participants, which introduces subjectivity and individual differences. Respondents may be influenced by social expectations or self-perception, challenging the reliability and accuracy of the results.

Cross-sectional measurement: This study employed a cross-sectional design, collecting data only at a specific time point, which does not allow for the observation of long-term relationships and dynamic changes between variables. Future research could employ longitudinal study designs to better understand the relationships and changes among these factors.

Potential confounding factors: Despite considering some potential confounding factors in the analysis, there may still be other unaccounted factors that could influence the results. For example, individual characteristics, work environment factors, and individual coping strategies may impact the results, which need to be further explored in future research.

Future studies could combine neural mechanisms, such as amygdala-prefrontal loop abnormalities, to explore biomarkers in the nurse population to develop precision intervention protocols.

### 4.3 Practical implications and recommendations

Given the high prevalence of PTSD among emergency department nurses and its strong associations with occupational burnout, maladaptive coping styles, low resilience, and reduced social support, the study underscores the urgent need for targeted, institution-level interventions. Hospital administrations and nursing managers should prioritize the implementation of structured support systems. These may include the creation of peer-led or professionally facilitated support groups, where nurses can process stress in a safe, nonjudgmental setting. Additionally, training programs in evidence-based coping strategies, such as cognitive-behavioral techniques or mindfulness-based stress reduction, could enhance positive coping and reduce emotional exhaustion. Initiatives to boost psychological resilience—such as resilience workshops, mentorship programs, or resilience coaching—can help staff adapt more effectively to acute stressors. Furthermore, efforts to increase access to and utilization of social support, both within and beyond the workplace (e.g., employee assistance programs, supervisor check-ins, team-based bonding activities), are essential in mitigating the impact of occupational trauma. These interventions should be tailored to the high-intensity, fast-paced nature of emergency departments and ideally be evaluated through future longitudinal studies to assess their long-term impact on mental health outcomes. Implementing stress management programs, reducing excessive workloads, and promoting a healthy work-life balance can lower burnout, which is a key risk factor for PTSD. Encouraging practices like yoga, meditation, and deep breathing exercises can help manage stress before it escalates into PTSD. Programs focused on reframing negative experiences and developing emotional regulation skills can enhance resilience, while Cognitive Behavioral Therapy can assist individuals in restructuring negative thoughts and building coping mechanisms to deal with traumatic memories.

## 5 Conclusion

The incidence of PTSD among emergency department nurses during the winter influenza season is a pressing concern that necessitates careful consideration. It is closely intertwined with factors such as burnout, psychological resilience, coping strategies, and social support. Addressing this issue promptly is of paramount importance. In the flu season, the workload of emergency department nurses should be rationally allocated, including temporary nursing staff and flexible scheduling system, so as to reduce the degree of nurse burnout and reduce the risk of PTSD.

## Data Availability

The original contributions presented in the study are included in the article/supplementary material, further inquiries can be directed to the corresponding author.
